# Survival of Patients with Solid Tumours and Sepsis Admitted to Intensive Care in a Tertiary Oncology Centre: A Retrospective Analysis

**DOI:** 10.1177/08850666241312621

**Published:** 2025-01-28

**Authors:** Sam S. Smith, Luke Edwards, Timothy Wigmore, Shaman Jhanji, David B. Antcliffe, Kate C. Tatham

**Affiliations:** 1 Division of Anaesthetics, Pain Medicine and Intensive Care, Department of Surgery and Cancer, Faculty of Medicine, 4615 Imperial College London, London, UK; 2 Department of Critical Care & Anaesthetics, 4970 The Royal Marsden Hospital NHS Foundation Trust, London, UK; 3 Division of Cancer Biology, 5053 Institute of Cancer Research, London, UK; 4 Department of Critical Care & Anaesthetics, The Royal Marsden Hospital NHS Foundation Trust, London, UK

**Keywords:** intensive care, cancer, sepsis, critical care

## Abstract

**Introduction:**

Sepsis is a life-threatening organ dysfunction caused by a dysregulated host response to infection. Patients with cancer are at risk of developing sepsis and requiring intensive care unit (ICU) admission. We aimed to assess survival of patients with a solid tumour admitted to ICU as an emergency with sepsis, and to identify predictors of 90-day survival at admission.

**Materials and Methods:**

We conducted a retrospective cohort survival analysis. We identified adults with a solid tumour admitted to ICU with sepsis between 01/01/2011 and 31/12/2020 at a tertiary oncology centre with two hospitals (London and Surrey, UK). We defined sepsis using the Sepsis-3 definition. The primary outcome was 90-day survival. We used the parametric accelerated failure time model for multivariate analysis to generate acceleration factors (AF).

**Results:**

625 patients were identified and the 90-day survival rate was 59.5%(353/593).Multivariate analysis identified the presence of localized (AF 0.13, 95% CI 0.06–0.25) or regionalized disease (AF 0.21, 95% CI 0.12–0.36) compared to distant metastatic disease, unplanned surgery on the day of admission (AF 0.15, 95% CI 0.07–0.31), lactate (AF 1.25 95% CI 1.15–1.35), Sequential Organ Failure Assessment Score (AF 1.19, 95% CI 1.12–1.27), previous radiotherapy (AF 1.89, 95% CI 1.14–3.125), previous systemic anti-cancer treatment (excluding hormonal therapy) (AF 1.49, 95% CI 0.93–2.38), bacteraemia (AF 0.47, 95% CI 0.27–0.81) and serum albumin (AF 0.94, 95% CI 0.91–0.98) as independent predictors of 90-day survival.

**Conclusions:**

This study of solid tumour patients admitted to ICU is one of the largest providing survival data to inform clinicians and patients. This data provides information on factors that should be considered when deliberating the possible outcome of ICU admission for a patient with solid malignancy and sepsis and highlights that the presence of cancer itself should not limit ICU admission for sepsis.

## Introduction

Sepsis is a life-threatening organ dysfunction caused by a dysregulated host response to infection.^1^ Sepsis is a common reason for admission to the intensive care unit (ICU), resulting in 30% of ICU admissions in the UK, and has a rising incidence.^2^ Septic shock is present in a subset of patients with sepsis, defined as those requiring vasopressors to maintain mean arterial pressure (MAP) over 65 mm Hg and lactate over 2.0 mmol/L despite adequate fluid resuscitation.^1^ A recent meta-analysis of 170 studies in critically unwell adults with sepsis identified 90-day survival for sepsis and septic shock as 68% and 61% respectively.^2^

Patients with cancer frequently develop sepsis with an incidence of 3.7% in the year following diagnosis, conferring a high mortality and leading to high healthcare costs.^3,4^ In ICU, these patients have previously been reported to have lower survival rates than non-cancer patients, with 90-day survival ranging from 28% to 72%, with the majority of studies identifying survival under 50%.^5–7^ Septic shock in cancer patients has even lower 90-day survival rates, with estimates varying between 29% and 49%. Interestingly reports of survival in patients with sepsis and solid tumours has been reported to be higher, lower or no different than haemato-oncology patients.^8–11^

Survival varying substantially between studies in cancer patients is likely due to multiple factors including varying definitions of sepsis and septic shock, improved management of sepsis over time e.g. with the adoption of the surviving sepsis campaign, varied geographical locations with differing clinical management strategies, heterogenous cohorts and low patient number. With regards to studies of sepsis in oncology patients, they often consist of mixed haematological and/or solid cancer cohorts.^13–15^

Previous studies, in mainly small cohorts, have made many associations with increased mortality in cancer patients with sepsis/septic shock. These include lactate, Acute Physiology and Chronic Health Evaluation (APACHE) II score, performance status, metastatic disease, need for renal replacement therapy and Sequential Organ Failure Assessment Score (SOFA) score.^6,9,16–20^ Bacteraemia has been associated with both increased and decreased mortality.^10,18,22^

In this study our objective was to identify 90-day survival rates for patients admitted to ICU with sepsis over a 10-year period, and the factors associated with 90-day survival, in patients with a history of solid organ cancer. We hypothesised that baseline clinical features would be associated with outcome.

## Materials and Methods

### Setting

The Royal Marsden Hospital NHS Foundation Trust is a tertiary referral centre consisting of two hospitals with 234 beds in total (London and Surrey, UK). Both sites have inpatient and outpatient oncology services, including an acute admission service which receives referrals from a hotline all outpatients can contact 24 h a day. There is no emergency department and no non-emergency cardiothoracic, neurosurgical or vascular surgical services. All ICU admissions are approved by an ICU consultant who is available 24 h a day.

### Study Design and Data Collection

This study was submitted to, and approved by, the Service Evaluation Board, Department of Research and Development, Royal Marsden NHS Foundation Trust (Study number: SE1140, Approval date: 13th April 2022). Informed consent for this is waived by our institution.

All emergency/unplanned admissions to intensive care within our trust were identified between first January 2011 and 31st December 2020. Patients admitted following planned non-emergency surgery were not included. Data were collected retrospectively from the ICU or trust electronic patient record system. Only the first emergency admission to critical care was considered. Where patients had not died by data collection (22nd June 2022) their last attendance at the hospital was used for censoring.

Patients admitted with sepsis were identified as those with a SOFA score of 2 or more and suspected infection (defined as antimicrobial administration within 24 h of admission to ICU and blood cultures sent within 2 days before or 2 days after admission to ICU), based on the sepsis-3 definition.^1^ The SOFA score was calculated using data from the first 24 h of admission to ICU. To avoid missing patients that met the sepsis-3 definition, we did not require documentation of sepsis in the patient notes. It was not possible to assess whether appropriate antimicrobials were administered.

Physiological data and blood results were extracted from the first 24 h of admission to ICU, from which the SOFA score was calculated. Organ support was recorded throughout ICU admission. Demographics and cancer diagnosis and treatment was extracted from data preceding ICU admission. We excluded hormonal therapies from our systemic anti-cancer treatment (SACT) category and we were unable to identify treatment intent or performance status prior to ICU admission.

Full details of data extraction are given in the Supplementary Methods. Septic shock on admission was defined as lactate over 2 mmol/L and the use of vasopressors in the first 24 h as per the Sepsis-3 definition^1^; we assumed adequate fluid resuscitation prior to the commencement of vasopressor as we did not have access to this data from before ICU admission.

### Statistical Analysis

Data were analysed using R version 4.2.1 using the RStudio interface. Raw survival was calculated excluding patients lost to follow up. We chose 90-day survival as our key outcome as we thought that more prolonged survival would be more likely to reflect the underlying cancer rather than the septic episode. Univariate analyses were conducted between patients known to be dead or alive at day 90, excluding patients lost to follow up. All values were considered in analysis; no outliers were excluded. We undertook univariate analysis using the variables shown in Table 2, Supplementary Table 2, Supplementary Table 4, Supplementary Table 5. We chose these variables as they include commonly collected demographic, intensive care and oncology data which has been collected in previous studies and were feasible to collect.

Multivariate analysis was undertaken using data available within 24 h of admission. We chose the variables listed in Supplementary Table 6 for multivariate analysis based on previous similar studies and to ensure that we included variables that provided unique information while limiting co-linearity; this reasoning is explained further in the supplementary methods. We excluded patients with in-situ disease on Surveillance, Epidemiology and End Results (SEER) summary staging from multivariate analysis due to their low number and the expectation that they did not have malignant disease. We also excluded patients with incomplete data. We found that the proportional hazards assumption was violated by our dataset so used the parametric accelerated failure time model for multivariate analysis. To enable inclusion of patients who died on day 0 they were recoded as dying on day 0.5. The generalised gamma distribution was used as it provided the best model according to Akaike information criterion (AIC). Forward selection was used to generate the best model according to AIC. We inputted unique variables to avoid co-linearity (eg as SOFA was included vasopressor use and respiratory support were not) which were related to the first 24 h of ICU admission (not the entire ICU stay). Further details on variable selection are explained in the supplementary methods. Inclusion of survival data for the underlying cancer to control for expected mortality from the cancer itself is explained in the supplementary methods. Accelerated failure time models do not generate hazard ratios but instead time ratios and acceleration factors. We have presented our findings as acceleration factors, which is a ratio of quantiles of survivor function. Like hazard ratios, a value over 1 corresponds to an expectation of increased time to the event in one group over another.

## Results

**Figure 1.**
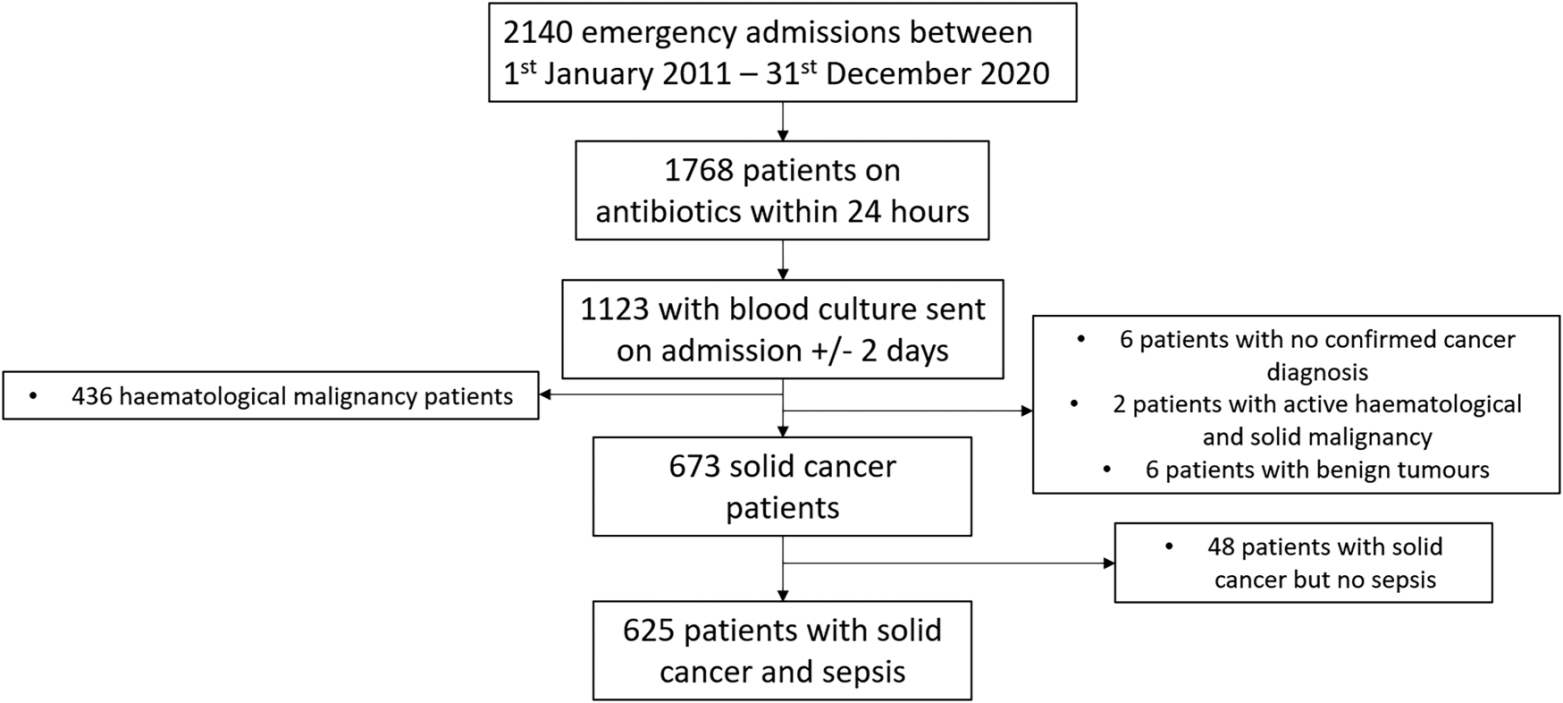
**Flow chart demonstrating selection of patients. Figure 1.** Flow chart demonstrating selection of emergency admissions to ICU with solid tumours and sepsis. 625 patients with solid tumours and sepsis were identified. All these patients were considered in survival analysis. 32 patients were lost to follow up by day 90 and hence were excluded from our univariate analysis which compared survivors and non-survivors at day 90. 620 patients were used for multivariate analysis. 2 patients were excluded as we were unable to collect complete data for all the variables considered in multivariate analysis. 3 patients were excluded as they had in situ cancer and this group was too small to compare to localised, regionalised or distant metastatic spread.

Patients with sepsis and solid organ malignancy were identified as shown in Figure 1. A total of 2140 emergency admissions to the critical care service were identified between first January 2011 and 31st December 2020. 625 patients with solid organ malignancy and sepsis were identified and were used for the overall survival analysis. Two patients had missing data and three had in situ cancer and were excluded from multivariate analysis. Median time to censoring was 145 days. Fifteen patients were lost to follow up by day 30, 32 patients by day 90 and 45 patients in total by day 180. The 593 patients who were not lost to follow up by day 90 were used for univariate analysis comparing day-90 survivors and non-survivors.

Patient characteristics are shown in Table 1. The median age was 64 years (interquartile range (IQR) 54–71) and 55.9% were male. The median ICU length of stay (LOS) was 3 days (IQR 2–7) and the median total hospital LOS was 21 days (IQR 11–36). The median length of stay in hospital before ICU admission was 4 days (IQR 1–10).

**Table 1.** Clinical Features of Patients Admitted to ICU with Solid Tumours and Sepsis.

**Variable**	**Median/n (IQR/%)**
**Demographics, n = 625**	
Age, years	64(54–71)
Male	349(55.9%)
Time to censoring, days	145(21–629)
Year	2016(2013–2018)
Hospital length of stay, days	21(11–36)
Length of stay before ICU admission, days	4(1–10)
ICU length of stay, days	3(2–7)
SEER summary stage	
In situ	3(0.5%)
Localized	123(19.7%)
Regionalized	160(25.6%)
Distant	339(54.2%)
Systemic Anti-Cancer Treatment	376(60.2%)
Radiotherapy	121(19.4%)
Surgery at any time before admission	374(59.8%)
Surgery on day of admission	83(13.3%)
Positive blood culture	132(21.1%)
Any positive culture	283(45.3%)
**First 24 h of admission, n = 625**	
Vasopressor use	259(41.4%)
Renal replacement therapy	43(6.9%)
Septic Shock	147(23.5%)
Neutropenia	57(9.1%)
Invasive Ventilation	156(25%)
NIV	77(12.3%)
High Flow Nasal Cannulae	144(23%)
SOFA	6(4–9)
APACHE II	19(15–24)
**Throughout admission, n = 625**	
Vasopressor use	332(53.1%)
Renal replacement therapy	80(12.8%)
Invasive Ventilation	210(33.6%)
NIV	112(18.0%)
High Flow Nasal Cannulae	216(34.6%)

Data on all Patients Admitted with Solid Tumours and Sepsis. (NIV = non-Invasive Ventilation, LOS = Length of Stay, SOFA = Sequential Organ Failure Assessment Score, SEER = Surveillance, Epidemiology and End Results, APACHE II = Acute Physiology and Chronic Health Evaluation II.).

The most common anatomical location of the malignancies that were included were lower gastrointestinal (16.0%), upper gastrointestinal (14.4%), gynaecological (10.9%), soft tissue sarcomas (7.5%), lung (6.6%) and pancreas (6.9%) (Supplementary Table 1). According to SEER summary staging, 19.7% had localized disease, 25.6% had regionalized disease and 54.2% had distant metastases (Table 1). In our cohort 60.2% of patients had received SACT (excluding hormonal therapy), 19.4% had received radiotherapy and 59.8% had undergone surgery. 13.3% of patients had undergone emergency surgery on the same day as their emergency admission to ICU (Table 1). Of those who had received SACT prior to ICU admission, 67.0% had received treatment within 30 days of ICU admission and 95.2% within 180 days of ICU admission. At admission, positive blood cultures were present in 21.1% of patients; 45.3% had a positive blood, sputum or urine culture. Microbiological results are shown in Supplementary Table 3 and 4.

The median SOFA score was 6 (IQR 4–9) and the median APACHE II score 19 (IQR 15–24). On ICU admission, 23.5% of patients had septic shock according to sepsis-3 definitions. 41.4% of patients required vasopressors within 24 h of ICU admission and 53.1% of patients received vasopressors at some point during their ICU admission. 25.0% of patients were intubated and mechanically ventilated by 24 h into ICU admission and 33.6% at some point during their ICU stay.

ICU survival was 84.8% and hospital survival 74.1% (Table 2). The 30, 90 and 180-day raw survival was 72.0%, 59.5% and 51.7% respectively. Considering only patients with septic shock, the 30, 90 and 180-day raw survival was 57.8%, 46.5% and 40.6% respectively. 90-day survival by cancer type is given in Supplementary Table 1. Assessing a longer time frame, the 1-year and 5-year survival for all patients was 38.7% and 8.1% respectively and for septic shock 32.6% and 6.3% respectively. 8.6% of patients were lost to follow up at one year and 21% at five years.

**Table 2.** Survival for patients admitted to ICU with solid tumours and sepsis.

**All Patients**	**Raw survival, n/% (% / 95% CI)**
ICU, n = 625	530(84.8%)
Hospital, n = 625	463(74.1%)
30 day, n = 610	439(72%)
90 day, n = 593	353(59.5%)
180 day, n = 580	300(51.7%)
365 day, n = 571	221(38.7%)
5 year survival, n = 494	40(8.1%)
365 day survival probability	41%(37%-45%)
5 year survival probability	18%(14%-21%)
**Patients with sepsis only**	
ICU, n = 478	423(88.5%)
Hospital, n = 478	373(78.0%)
30 day, n = 463	354(76.5%)
90 day, n = 449	286(63.7%)
180 day, n = 437	242(55.4%)
365 day, n = 430	175(40.7%)
5 year survival, n = 368	32(8.7%)
365 day survival probability	43%(39%-48%)
5 year survival probability	19%(15%-24%)
**Patients with septic shock only**	
ICU, n = 147	107(72.8%)
Hospital, n = 147	90(61.2%)
30 day, n = 147	85(57.8%)
90 day, n = 144	67(46.5%)
180 day, n = 143	58(40.6%)
365 day, n = 141	46(32.6%)
5 year survival, n = 126	8(6.3%)
365 day survival probability	34%(27%-43%)
5 year survival probability	12%(7.2%-21%)

Survival Data for Patients Admitted with Solid Tumours and Sepsis. the First Group is all Patients and the Second Group is a Subset of the First; Those with Septic Shock. Survival Probabilities Were Generated Using Kaplan Meier Methods.

Of patients with distant metastatic disease 90-day survival was 44.0% (144/327). Of these patients, 81 had septic shock. Their 90-day survival was 28.4% (23/81). In contrast, of patients with only regional cancer spread, 90-day survival was 76.0% (117/154). 31 of these patients had septic shock and their 90-day survival was 61.3% (19/31).

### Risk Factors

#### Univariate Analysis

**Figure 2.**
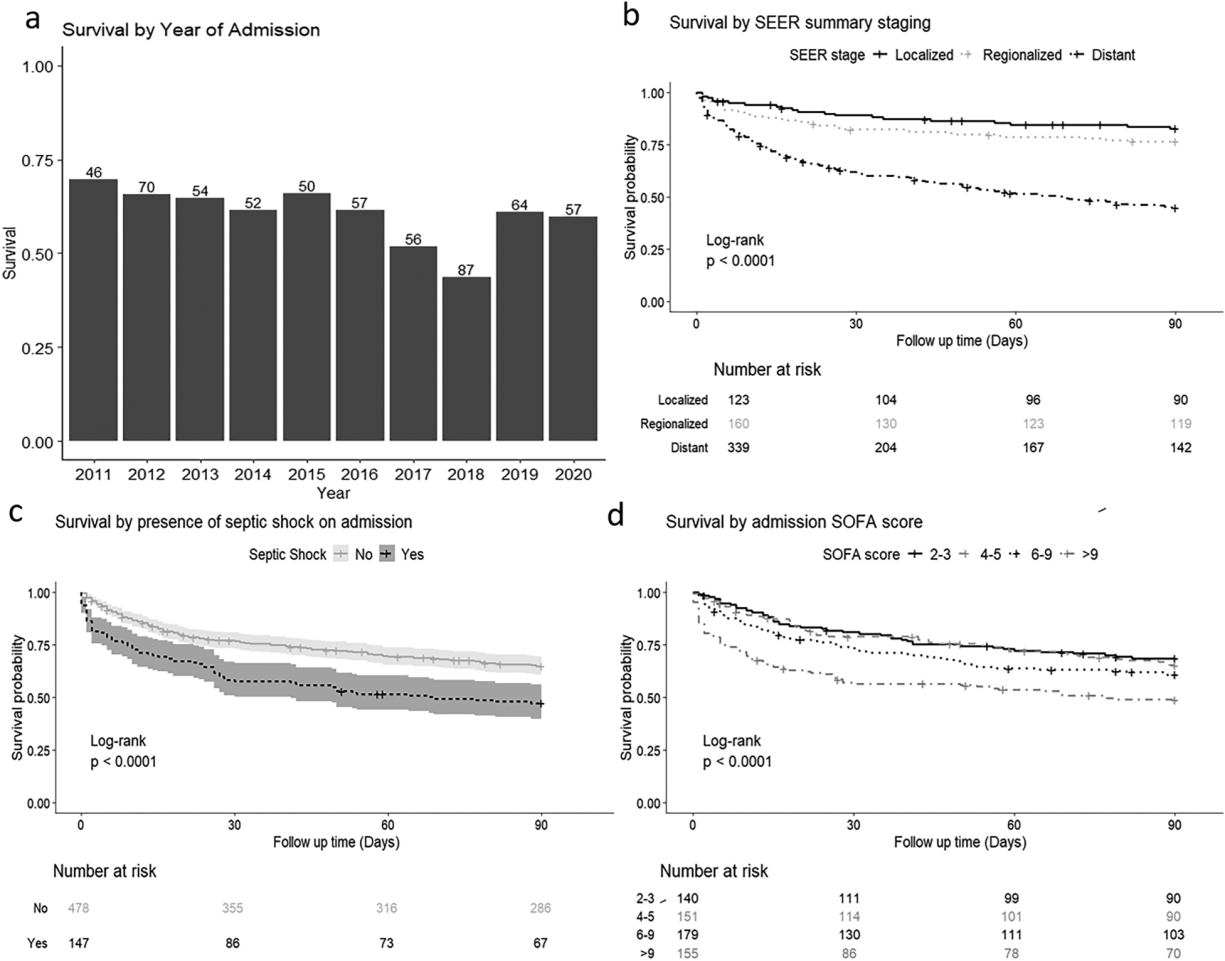
**90 day survival stratified by various categorical variables. Figure 2.** 90 day survival stratified by various categorical variables. a) 90-day survival by year of admission to ICU. Survival is given as a proportion on the y axis. The number of patients admitted with sepsis in each year is shown above each column. There is no trend in survival over the 10 years studied with no statistically significant change (p = 0.085). b) 90-day survival base on SEER summary stage. Distant metastases have worse mortality then regionalized or localized disease (p < 0.0001). c) 90-day survival by the presence or absence of septic shock on admission. Patients with septic shock have significantly worse survival (p < 0.0001). d) 90-day survival by admission SOFA score. Increasing SOFA score confers a significantly increased risk of mortality (p < 0.0001). Statistical significance was tested using the chi squared test in a and the log rank test in b-d. (SOFA = Sequential Organ Failure Assessment Score, SEER = Surveillance, Epidemiology and End Results.).

Univariate analyses identified that lactate, albumin, SOFA score, APACHE, renal replacement therapy, septic shock (Figure 2c), neutropenia, SEER summary stage (Figure 2b), underlying diagnosis, SACT, underlying cancer and surgery were significantly different in day-90 non-survivors compared to survivors (Table 3, Supplementary Table 1, 2, 4). Of note, year of admission (Figure 2a), vasopressor use, respiratory support, radiotherapy and microbiological results were not significantly different between survivors and non-survivors at day 90 (Table 3, Supplementary Table 2, 4). A SOFA score of over 9 was associated with less than 50% survival by day 90 (Figure 2d). The renal, liver and coagulation components of the SOFA score were significantly different in survivors and non-survivors at day 90 whereas the respiratory, cardiovascular or neurological components were not (Supplementary Table 5). Sensitivity analyses are described in the supplement.

**Table 3.** Univariate analysis comparing survivors (n = 353) and non-survivors (n = 240) at day 90.

Variable	Day 90 survivors n = 353, median or n (IQR or %)	Day 90 non-survivors n = 240, median or n (IQR or %)	p value
**Demographics**			
Gender; n(%)			0.433
Male	201(56.9)	128(53.3)	
Age; years (IQR)	63(52–71)	64(55–71)	0.299
ICU LOS; days(IQR)	4(2–8)	3(2–6)	0.002
SEER Summary staging category; n(%)			<0.001
In situ	2(0.6%)	0(0%)	
Localised	90(25.5%)	20(8.3%)	
Regional spread	119(33.7%)	37(15.4%)	
Distant metastases	142(40.2%)	183(76.3%)	
Systemic Anti-Cancer Treatment; n(%)	191(54.1%)	174(72.5%)	<0.001
Radiotherapy; n(%)	62(17.6%)	54(22.5%)	0.167
Surgery at any time before admission; n(%)	249(70.5%)	103(42.9%)	<0.001
Surgery on day of admission; n(%)	65(18.4%)	15(6.3%)	<0.001
**Within 24 h of ICU admission**			
SOFA score (IQR)	5(3–9)	7(4–10)	<0.001
APACHE II score (IQR)	18(15–23)	20(15–25)	0.013
Septic shock; n(%)	67(19%)	77(32.1%)	<0.001
Vasopressor use; n(%)	136(38.5%)	109(45.4%)	0.112
Renal replacement therapy; n(%)	8(2.3%)	32(13.3%)	<0.001
Invasive ventilation; n(%)	92(26.1%)	58(24.2%)	0.671
Lactate concentration, mmol/L*; median(IQR)	1.5(1–2.4)	2.2(1.5–3.8)	<0.001
Albumin concentration, g/L; median(IQR)	22(19–26)	21(18–24)	<0.001
Neutropenia; n(%)	24(6.8%)	30(12.5%)	0.037
**At any time during ICU admission**			
Vasopressor use; n(%)	177(50.1%)	139(57.9%)	0.075
Renal replacement Therapy; n(%)	24(6.8%)	52(21.7%)	<0.001
AKI; n(%)	220(62.3%)	162(67.5%)	0.228
IV ; n(%)	127(36%)	77(32.1%)	0.373

Continuous Variables Were Compared Using Wilcoxon Rank sum Test. Categorical Variables Were Compared Using Chi Squared or Fisher's Exact Test. (AKI = Acute Kidney Injury, LOS = Length of Stay, SOFA = Sequential Organ Failure Assessment Score, SEER = Surveillance, Epidemiology and End Results, APACHE II = Acute Physiology and Chronic Health Evaluation II.)). * = Missing Data for 2 Patients.

#### Multivariate Analysis

Multivariate analysis was conducted with 27 more patients than univariate analysis as this model is able to consider patients lost to follow up. Two patients with incomplete data and three patients with in-situ disease were excluded from analysis. Forward selection identified the factors in Table 4 in the best model of 90-day survival. The variables considered in forward selection are shown in Supplementary Table 6.

**Table 4.** Multivariate model results of the best predictors of 90-day survival.

Covariate		Acceleration Factor	95% CI	p value
**SEER Summary Stage**	Distant	1.0	-	-
	Localised	0.13	(0.06–0.25)	<0.001
	Regionalised	0.21	(0.12–0.36)	<0.001
**SOFA**		1.19	(1.12–1.27)	<0.001
**Lactate**		1.25	(1.15–1.35)	<0.001
**Surgery on day of admission**	No	1.0	-	-
	Yes	0.15	(0.07–0.31)	<0.001
**Albumin**		0.94	(0.91–0.98)	0.001
**Bacteraemia**	No	1.0	-	-
	Yes	0.47	(0.27–0.8)	0.003
**Radiotherapy**	No	1.0	-	-
	Yes	1.89	(1.14–3.125)	0.007
**National one year survival of the underlying cancer**		0.99	(0.98–1.00)	0.009
**Age**		1.02	(1.00–1.04)	0.010
**Medical Cancer Treatment**	No	1.0	-	-
	Yes	1.49	(0.93–2.38)	0.048

Accelerated Failure Time Multivariate Model Identifying the Best Predictors for 90-day Survival. Acceleration Factors are Similar to Hazard Ratios. A Higher Value Indicates an Expected Shorter Event Time; Therefore Acceleration Factors Over 1 Indicate Worse 90 day Survival. for Example, no Medical Cancer Treatment Provides the Reference Acceleration Factor of 1. the Presence of Medical Cancer Treatment has an Acceleration Factor of 1.49, Indicating That Receiving Medical Cancer Treatment Corresponds to a Shorter Event Time (Time to Death) Compared to no Medical Cancer Treatment. P-Values Were Calculated Using the Wald Test. (SOFA = Sequential Organ Failure Assessment Score, SEER = Surveillance, Epidemiology and End Results.).

Independently, SEER summary stage was the strongest predictor of survival, with localized and regional disease having an acceleration factor (AF) of 0.13 (95% CI 0.06–0.25) and 0.21 (95% CI 0.12–0.36) respectively compared to distant spread of disease. Increasing SOFA and lactate values were also significantly and independently associated with worse survival. Surgery on the day of admission was independently associated with improved survival (AF 0.15 95% CI 0.07–0.31) while SACT (AF 1.49 95% CI 0.93–2.38) or radiotherapy (AF 1.89 95% CI 1.14–3.125) at any time before admission were independently associated with worse survival. Bacteraemia and increased albumin were associated with improved survival, while increasing age and reducing national survival of the underlying cancer were all associated with worse survival. Of note, including gender, year of admission or renal replacement therapy did not improve the model. Sensitivity analyses altering calculation of the respiratory SOFA score and inclusion of APACHE score II did not change our initial findings (Supplementary table 7). In addition, exclusion of the variable national one-year survival of the underlying cancer did not substantially alter the results of our multivariate analysis (Supplementary table 8). Furthermore, we undertook a sensitivity analysis considering patients not lost to follow up by day 90. We conducted a binary logistic regression with stepwise selection to identify the best predictors of death or survival at day 90 (supplementary table 9). This selected the same variables in Table 4 in addition to year of admission and renal replacement therapy on admission.

## Discussion

We found that 90-day survival for solid cancer patients admitted to the ICU with sepsis was 59.5% and for those with septic shock it was lower (46.5%). The presence of distant metastases, increasing lactate and SOFA scores, previous radiotherapy or SACT and hypoalbuminaemia were independently associated with reduced 90-day sepsis survival. Surgery on the day of admission and bacteraemia were associated with improved 90-day survival.

These survival rates are higher than found in many studies investigating cancer associated sepsis and comparable to survival rates reported in patients without cancer.^2^ There are multiple possible explanations for these findings: we utilised the more recent Sepsis-3 definition which has been found to be a more liberal sepsis definition than Sepsis-2 in some populations,^22^ our patient population was less unwell than patients in other studies assessing both cancer and non-cancer populations with lower SOFA and APACHE II scores on admission and a high proportion of patients not receiving organ support within 24 h of admission (41.6%). Another factor could be that we had a relatively low proportion of patients with distant metastatic disease and we found that the presence of distant metastases significantly increased the risk of death. For example, Cuenca et al identified 90-day survival of patients with septic shock and metastatic disease as 19.7%^17^ and we identified 90-day survival of patients with septic shock limited to those with distant metastatic disease of 28.4%. In contrast, we found patients with regional spread of disease had 90-day survival of 61.3%. Nevertheless, a 90-day survival rate of 59.5% is encouraging and may represent the improving treatment of sepsis both in and out of ICU.

Some studies have only considered patients with “active” cancer excluding those with successful resections^5^ whereas our population included patients with successful resections. However, 54.2% of our population had metastatic disease who would all have “active cancer”. Another reason for possible differences was that we had a low proportion of patients with lung cancer, as compared to Cuenca et al^17^ and it has previously been identified that patients with lung cancer and sepsis have worse survival than patients with other tumour types.^23^ Furthermore, previous studies have been conducted in a range of geographical locations that may have differing cancer and sepsis care.^8,17,19,24–26^

In multivariate analysis, inclusion of the underlying national survival for each cancer type enabled identification of risk factors while accounting for the expected survival of the underlying cancer. Distant metastases conferred a substantially greater mortality risk, confirming previous findings.^19^ Unsurprisingly, higher SOFA scores and lactate were associated with reduced survival, as has been found in previous studies in patients with cancer and sepsis.^7,8,10,16^ Interestingly, the only other biochemical marker associated with survival was albumin, with a lower value conferring reduced survival. This has been found in critically ill cancer patients and non-cancer sepsis patients but not specifically those with sepsis and cancer.^27,28^ Neutropenia was not associated with reduced survival, as has been previously reported.^17^

In terms of cancer treatment, radiotherapy and SACT at any time before admission were independently associated with reduced survival even when accounting for SEER summary stage. Previous studies have identified the association of palliative radiotherapy with mortality in ICU, but not specifically in sepsis.^29^ As we were unable to identify whether patients were receiving treatment with palliative or curative intent further investigation is necessary to identify whether disease status, treatment intent or the treatment itself is responsible for the association between radiotherapy and SACT and survival in our study. Surgery on the day of admission was associated with improved survival. There are many potential reasons for this finding in our population, firstly surgery enables effective source control of infection and so could lead to more effective treatment, however, only the fitter patients would be candidates for surgery so this could represent selection bias.

Bacteraemia was independently associated with improved survival. Previous studies have found varying associations between bacteraemia and survival in sepsis in either patients with or without cancer.^18,25,30^ Growth of a specific pathogen enables appropriate antibiotic selection and potentially more effective bacterial clearance. However, it may also be the case that some patients who are culture negative had diagnoses other than sepsis so failed to respond to treatment. The rate of misdiagnosis of infection could be particularly high for a population with cancer where usual signs of infection (eg fever) may be disease related and/or reactions to cancer treatment.

Our retrospective analysis has several strengths. Our patient cohort is larger than many studies, with 593 patients followed to day 90. In addition, we only assessed patients with solid tumours given the treatment differences in haematological cancers. We used only variables around admission in multivariate analysis to enable comparison with individuals who have short stays in ICU and provide information for future decision making. However, this study has several limitations which we attempted to minimise. Firstly, this study was a retrospective analysis of a single specialist cancer centre. In addition, the specialist nature of our institution limits the generalisability of our study to cancer patients admitted to ICUs in non-cancer centres. Furthermore, our centre does not undertake thoracic surgery and therefore lung cancer patients are under-represented. We attempted to limit bias and user error by extracting the majority of data computationally; however manual extraction of data required some utilisation of patient electronic records. Performance status and treatment intent are important factors in the decision to admit to ICU and resulting survival ^30,31^. Unfortunately, we were not able to include performance status and treatment intent as we found that this information was variably recorded and often uncertain at the time of ICU admission. However, it is unlikely that patients with no further treatment options would be considered for ICU admission. We were also unable to include patients with sepsis who were not admitted to ICU. Another potential limitation is that we assumed a baseline SOFA score of zero because we were unable to access the complete patient record, prior to the ICU admission. Therefore, patients may not have had a true change in SOFA score of two or more. Assessment of antimicrobials appropriateness and timing, and the adequacy of fluid replacement prior to vasopressor initiation, lay outside the remit of this study as these are both difficult to judge accurately in retrospective analysis but are also important considerations. Our study considered patients admitted to ICU over a ten-year time period. This could be considered a limitation, as treatment for many metastatic cancers have changed dramatically over this period. However, we found no significant change in survival based on year of admission despite improving overall cancer survival in this time period.^26^ The reasons underlying this are unclear. While speculative, it may be that due to improving cancer treatments patients who have more advanced disease were considered for ICU admission in later years whereas they were not in earlier years. However, this may also reflect that there were only around 60 admissions each year.

## Conclusions

This retrospective study of patients with solid tumours admitted to ICU with sepsis is one of the largest of its kind with the longest follow up period, providing survival data to inform clinicians and patients alike. We found that 90-day survival was 59.5%, which is higher than many previous studies. Risk factors for worse 90-day survival were the presence of distant metastases, increasing lactate and SOFA scores, previous radiotherapy or SACT and hypoalbuminaemia. Surgery on the day of admission and bacteraemia were associated with improved 90-day survival. Decisions to admit patients with sepsis to the ICU should be guided by the likelihood of surviving sepsis and not solely the presence or absence of solid tumours. Further research, particularly at the point of referral to ICU/ intensive care outreach, is necessary to guide this decision making. Inclusion of performance status in future research would be beneficial.

## Supplemental Material

sj-docx-1-jic-10.1177_08850666241312621 - Supplemental material for Survival of Patients with Solid Tumours and Sepsis Admitted to Intensive Care in a Tertiary Oncology Centre: A Retrospective Analysis Supplemental material, sj-docx-1-jic-10.1177_08850666241312621 for Survival of Patients with Solid Tumours and Sepsis Admitted to Intensive Care in a Tertiary Oncology Centre: A Retrospective Analysis by Sam S. Smith, Luke Edwards, Timothy Wigmore, Shaman Jhanji, David B. Antcliffe and Kate C. Tatham in Journal of Intensive Care Medicine

sj-docx-2-jic-10.1177_08850666241312621 - Supplemental material for Survival of Patients with Solid Tumours and Sepsis Admitted to Intensive Care in a Tertiary Oncology Centre: A Retrospective Analysis Supplemental material, sj-docx-2-jic-10.1177_08850666241312621 for Survival of Patients with Solid Tumours and Sepsis Admitted to Intensive Care in a Tertiary Oncology Centre: A Retrospective Analysis by Sam S. Smith, Luke Edwards, Timothy Wigmore, Shaman Jhanji, David B. Antcliffe and Kate C. Tatham in Journal of Intensive Care Medicine
