# Supplementary material for: Survival of Patients with Solid Tumours and Sepsis Admitted to Intensive Care in a Tertiary Oncology Centre: A Retrospective Analysis
Source: J Intensive Care Med. 2025 Jan 28;40(6):642–50. doi: 10.1177/08850666241312621 (PMC12095884; doi:10.1177/08850666241312621)
Supplement: sj-docx-1-jic-10.1177_08850666241312621 - Supplemental material for Survival of Patients with Solid Tumours and Sepsis Admitted to Intensive Care in a Tertiary Oncology Centre: A Retrospective Analysis [file sj-docx-1-jic-10.1177_08850666241312621.docx]

**Supplementary Methods and Tables**

Survival of patients with solid tumours and sepsis admitted to intensive care in a tertiary oncology centre: a retrospective analysis

Sam Smith^1,2^, Luke Edwards^2^, Timothy Wigmore^2^, Shaman Jhanji^2,3^, David B. Antcliffe*^1^, Kate C. Tatham*^1,2^

**Contents**

Supplementary methods

Supplementary Results

Supplementary table 1. Types of cancer in patients included in this study.

Supplementary table 2. Supplementary univariate analyses between survivors and non-survivors at day 90.

Supplementary table 3. Microbiological results from admission.

Supplementary table 4. Positive microbiology in survivors and non-survivors at day 90.

Supplementary table 5. Supplementary information on APACHE II and SOFA scores in survivors and non-survivors at day 90.

Supplementary table 6. Variables considered in multivariate analysis.

Supplementary table 7. Sensitivity analyses.

Supplementary table 8. Multivariate sensitivity analysis.

Supplementary table 9. Sensitivity analysis: binary logistic regression.

Supplementary references

**Supplementary methods**

**Identification of sepsis – additional information**

We assumed a baseline SOFA score of 0 as we did not have access to ward observations and that the patients were admitted to ICU due to an acute change in their physiology. We were unable to identify the time of commencement of antibiotics outside critical care due to use of paper drug charts on the wards. We used commencement of antibiotics with receipt of blood cultures between 2 days before and 2 days after ICU admission as an indicator of clinical suspicion of infection. We required receipt of blood cultures to exclude patients receiving prophylactic antimicrobials.

**Calculation of SOFA score**

We calculated the admission SOFA score by considering all data from the 24 hours following ICU admission. For the respiratory component we calculated the PaO2/FiO2 (PF) ratio through identifying PaO2 from the first 24 hours and the closest FiO2 reading. We identified and excluded venous gas samples defined as a gas saturation at least 10% lower than the closest peripheral saturation reading. We only included patients undergoing invasive mechanical ventilation as having respiratory support for SOFA calculation. Where patients did not have a PF ratio within 24 hours following admission, we assigned a score of 0 under the assumption that patients who did not have an arterial gas were unlikely to have respiratory compromise. We did not use SaO2/FiO2 (SF) ratio due to a lack of validation of this method as a surrogate for PF ratio^1^.The initial description of SOFA did not specify use of GCS with or without sedation; therefore we used the lowest GCS^2^. We attempted to identify the GCS before sedation but a lack of accuracy in data entry time into the electronic health records meant we did not have confidence that we were truly identifying the GCS before sedation. In line with previous publications, where SOFA component data were missing we assumed a value of 0^3^. Where urine output was not measured we assumed over 500ml of urine was passed. Where patients had length of stay of under 24 hours we extrapolated urine output to a length of stay of 24 hours. Where blood results were missing in the 24 hours following ICU admission, the result closest to this time period was used.

**Data Extraction**

The ICU has an electronic patient record (EPR) system where physiological data, medical notes, medication and laboratory results are recorded contemporaneously. The trust uses a custom EPR system which contains records on all interactions with the trust (inpatient or outpatient), laboratory results and demographic data. Data from the EPR is stored in a SQL database in Microsoft Access and was extracted using SQL queries.

The following data were extracted from the first 24 hours following ICU admission: minimum GCS, minimum PF ratio, mode of ventilatory support, minimum mean arterial blood pressure, maximum vasopressor rate, minimum platelet count, maximum creatinine, maximum bilirubin, maximum lactate, minimum neutrophil count, minimum lymphocyte count, minimum haemoglobin, minimum glucose, maximum C-reactive protein (CRP) and APACHE II score (including the proportion of missing variables required for calculation of APACHE II). The following demographic information was collected: the date and time of admission and discharge from ICU, ICU location, age and gender. The following data was collected throughout the entire admission: vasopressor use at any time, renal replacement therapy at any time, acute kidney injury (AKI; defined as variation in creatinine by over 26 umol/L during critical care stay) and mode of ventilation. Neutropenia was defined as a neutrophil count of under 1.5 x10^9^/L.

The following information was extracted from the EPR database computationally: previous surgery, previous radiotherapy, previous medical cancer treatment, cancer diagnosis, date of admission and discharge from hospital, and the most recent clinic attendance. The following data was extracted from the EPR database computationally within 2 days before and 2 days after admission: blood culture receipt, blood culture results, urine culture results, stool culture results and sputum culture results. PCR results (from any sample, principally blood or respiratory swab), beta-d-glucan and galactomannan were collected from 1 week before and 2 weeks after admission given that these samples are less commonly collected at admission. Summary staging according to the Surveillance, Epidemiology and End Results program was manually identified by one author through review of clinical notes, histopathology, imaging and multi-disciplinary team meetings available at the time of admission to ICU. Site of distant metastasis was not collected.

Extreme values were manually reviewed to ensure accuracy and correct erroneous values. Missing values were manually reviewed to confirm the data was truly missing. The nearest blood result to the missing value was manually identified if no value existed within 24 hours following ICU admission and used in its place.

**Data Analysis**

Data was analysed using RStudio version 2022.02.3+492 "Prairie Trillium" , 2022-05-20) for Windows and R version 4.2.1 (2022-06-23) -- "Funny-Looking Kid". The following packages were used: tidyverse, survival, survminer, XLConnect, psych, rms, pec, flexsurv and finalfit.

Where continuous variables were grouped as categorical variables for data analysis or representation 3 or 4 approximately evenly sized groups were used.

Continuous variables were analysed using Wilcoxon rank sum test with continuity correction because no continuous data was normally distributed according to the Shapiro-Wilk statistic. Categorical variables were analysed according to their contingency table. Where the count was too low for Pearson’s Chi-squared to be accurate (expected count under 5 in one cell) Fisher’s Exact Test for count data was used. Where the exact test was unable to be carried out due to insufficient computational power a simulated p value based on 10 million simulations was used. Otherwise Pearson’s Chi-Squared test was used; Yates’ continuity correction was applied for all 2X2 contingency tables. Kaplan Meier survival curves were created using the survival package in R. Curves were compared using the log rank test.

**Multivariate analysis**

The categorical variable year was grouped into 3 groups of roughly equal size to limit the number of covariates in regression and account for possible changes in treatment and hence survival over time. Therefore, the only variables considered in univariate analysis and not considered for multivariate analysis are listed below, with the reasons for their exclusion described. ICU LOS (not available on admission to ICU), surgery at any time before admission (patients may have had surgery at different centres and surgery years before ICU admission is unlikely to be relevant, in addition inclusion of surgery on the day of admission creates co-linearity), APACHE II (high amount of missing data), vasopressor use (co-linearity with SOFA score) and invasive ventilation (co-linearity with SOFA score). We did not consider information after 24 hours of ICU admission as we did not think it would be beneficial for our multivariate analysis.

We attempted to account for the mortality associated with the type of cancer in the multivariate analysis by including the one year survival rate of the underlying cancer in the multivariate model. We were unable to include each individual cancer type as the number of variables we would have had to included in the multivariable analysis would have been too great. We used national one year survival data for different cancers from years 2011-2015 within the National Cancer Registration and Analysis Service Routes to Diagnosis 2006-2016 workbook for this purpose ^4^. No large dataset contained data on survival of gastro-esophageal cancer so its survival was calculated as the average of gastric and esophageal cancer. The period of 2006-2016 was used as no other dataset contained survival data on all the cancer types within the timeframe of this study. We chose one year survival data for several reasons. Firstly, no large dataset provided 90 day survival data. Furthermore, this one year survival dataset considered data from the time of their diagnosis onwards, and the majority of patients admitted to ICU were not diagnosed with cancer immediately before their ICU admission.

**Sensitivity analysis**

We undertook sensitivity analysis to confirm our assumption that allocating a respiratory score of 0 to patients without a PF ratio was valid by comparing patients with a respiratory score of 0 with and without a PF ratio. We also undertook sensitivity analysis to assess whether the missing APACHE II variables impacted our analysis by only considering individuals without any missing variables. We also undertook sensitivity analysis of our final multivariate model excluding national one year survival of the underlying cancer. Finally, we conducted a sensitivity analysis considering only patients not lost to follow up by day 90. We undertook a binary logistic regression using stepwise selection considering the variables listed in supplementary table 6 to generate an alternative model for survival at day 90.

**Supplementary Results**

Sensitivity analysis

In SOFA score calculation, a respiratory score is assigned based on the PF ratio which cannot be calculated without arterial blood sampling. We assigned individuals without arterial blood samples a respiratory SOFA of 0 and undertook sensitivity analysis to assess our assumption that these patients had normal respiratory physiology. Sensitivity analysis separating patients allocated a respiratory SOFA score of 0 into those with and without a PF ratio in the first 24 hours of ICU admission revealed no difference in 90 day survival between these groups (Supplementary Table 7). Furthermore, despite missing variables for APACHE II calculation being found more frequently in non-survivors (Supplementary Table 4) assessment of the APACHE II scores in those patients with no missing variables confirmed only a slight increase in the median APACHE II score (from 20 to 21) in the patients who died by day 90 and in the patients who were alive at day 90 (from 18 to 19) (Supplementary Table 6).

We included national one year survival of underlying cancer in our multivariate model to attempt to account for different prognoses between different cancers. We repeated our final multivariate model excluding national one year survival of underlying cancer as a sensitivity analysis and found that the time ratios and p values were very similar (Supplementary Table 8).

**Supplementary tables**

**Supplementary table 1. Types of cancer in patients included in this study.**

| **Cancer** | **All patients**  **(n=625), n (%)** | **National one year survival, %** | **Day 90 survivors (n= 353), n(as % of cases of each cancer at day 90)** | **Day 90 non-survivors, (n= 240), n (as % of cases of each cancer at day 90)** |  |
| --- | --- | --- | --- | --- | --- |
| THYROID | 1(0.2%) | 93.4 | 0(0.0%) | 1(100%) |  |
| LUNG | 41(6.6%) | 36.2 | 7(20.6%) | 27(79.4%) |  |
| PROSTATE | 21(3.4%) | 95.8 | 5(25%) | 15(75%) |  |
| CANCER UNKNOWN PRIMARY | 16(2.6%) | 18.7 | 4(26.7%) | 11(73.3%) |  |
| BRAIN | 3(0.5%) | 41.3 | 1(33.3%) | 2(66.7%) |  |
| MESOTHELIOMA | 3(0.5%) | 39 | 1(33.3%) | 2(66.7%) |  |
| BREAST | 34(5.4%) | 96.2 | 15(45.5%) | 18(54.5%) |  |
| GASTRIC | 24(3.8%) | 43.2 | 11(45.8%) | 13(54.2%) |  |
| PANCREATIC | 43(6.9%) | 21.4 | 19(46.3%) | 22(53.7%) |  |
| COLORECTAL | 16(2.6%) | 76 | 7(50%) | 7(50%) |  |
| HYPOPHARYNX | 2(0.3%) | 58.8 | 1(50%) | 1(50%) |  |
| ORAL | 8(1.3%) | 78.3 | 4(50%) | 4(50%) |  |
| VAGINA | 3(0.5%) | 68.4 | 1(50%) | 1(50%) |  |
| ESOPHAGEAL | 35(5.6%) | 43.9 | 20(58.8%) | 14(41.2%) |  |
| BILIARY | 30(4.8%) | 26.1 | 18(60%) | 12(40%) |  |
| UTERUS | 21(3.4%) | 90 | 12(63.2%) | 7(36.8%) |  |
| CERVICAL | 11(1.8%) | 87.5 | 7(63.6%) | 4(36.4%) |  |
| SKIN | 17(2.7%) | 97.3 | 11(64.7%) | 6(35.3%) |  |
| BONE | 3(0.5%) | 82.5 | 2(66.7%) | 1(33.3%) |  |
| LIVER | 8(1.3%) | 39.3 | 4(66.7%) | 2(33.3%) |  |
| NONSPECIFIC HEAD & NECK | 3(0.5%) | 75.3 | 2(66.7%) | 1(33.3%) |  |
| COLON | 53(8.5%) | 76 | 35(67.3%) | 17(32.7%) |  |
| OROPHARYNX | 21(3.4%) | 83.2 | 13(68.4%) | 6(31.6%) |  |
| OVARIAN | 32(5.1%) | 74.3 | 22(68.8%) | 10(31.2%) |  |
| RENAL | 19(3.0%) | 76.6 | 12(70.6%) | 5(29.4%) |  |
| SOFT TISSUE SARCOMA | 47(7.5%) | 76.9 | 32(71.1%) | 13(28.9%) |  |
| LARYNX | 7(1.1%) | 83.7 | 5(71.4%) | 2(28.6%) |  |
| TESTICULAR | 16(2.6%) | 98.3 | 11(73.3%) | 4(26.7%) |  |
| SMALL INTESTINE | 16(2.6%) | 67.8 | 13(81.3%) | 3(18.7%) |  |
| RECTAL | 28(4.5%) | 76 | 23(85.2%) | 4(14.8%) |  |
| BLADDER | 23(3.7%) | 71.2 | 18(85.7%) | 3(14.3%) |  |
| GASTRO-ESOPHAGEAL JUNCTION | 15(2.4%) | 43.55 | 13(86.7%) | 2(13.3%) |  |
| ANUS | 3(0.5%) | 85.1 | 2(100%) | 0(0%) |  |
| NON SPECIFIC URINARY | 1(0.2%) | 69.6 | 1(100%) | 0(0.0%) |  |
| VULVA | 1(0.2%) | 83.9 | 1(100%) | 0(0.0%) |  |

Supplementary table 1. Types of cancer in patients admitted with sepsis and solid tumours. Separated into all patients, survivors and non-survivors at day 90. In survivors and non-survivors at day 90, the % is calculated by considering only individuals with that specific cancer type who were followed up to day 90. Fisher’s Exact test with simulated p value showed a significant difference between the cancers in survivors and non-survivors (p < 0.001 , Fisher’s Exact Test for Count Data with simulated p-value). National one year survival of the cancer type is also presented. Colon and rectal cancer were grouped together as colorectal in survival. Gastro-eophageal survival is the average of gastric and esophageal survival.

**Supplementary table 2. Supplementary univariate analyses between survivors and non-survivors at day 90.**

|  | **Variable** | **Day 90 survivors, n = 353, median or n (IQR/% of total in each row)** | **Day 90 non-survivors, n = 240, median or n (IQR/% of total in each row)** | | **p value** |
| --- | --- | --- | --- | --- | --- |
| **Demographics** | |  |  | |  |
|  | Year |  |  | | 0.085 |
|  | 2011 | 32(69.6%) | 14(30.4) | |  |
|  | 2012 | 46(65.7%) | 24(34.3%) | |  |
|  | 2013 | 35(64.8%) | 19(35.2%) | |  |
|  | 2014 | 32(61.5%) | 20(38.5%) | |  |
|  | 2015 | 33(66%) | 17(34%) | |  |
|  | 2016 | 35(61.4%) | 22(38.6%) | |  |
|  | 2017 | 29(51.8%) | 27(48.2%) | |  |
|  | 2018 | 38(43.7%) | 49(56.3%) | |  |
|  | 2019 | 39(60.9%) | 25(39.1%) | |  |
|  | 2020 | 34(59.6%) | 23(40.4%) | |  |
|  | Hospital LOS before ICU, days | 4(1-10) | 3(1-10) | | 0.566 |
|  | Hospital LOS total, days | 23(14-42) | 18(9-30) | | < 0.001 |
|  | Days to censoring | 522(248-1078) | 14(5-39.3) | | <0.001 |
| **Within 24 hours of admission** | |  |  | |  |
|  | Nasal high flow oxygen | 82(59.9%) | 55(40.1%) | | 1 |
|  | NIV | 42(56.8%) | 32(43.2%) | | 0.695 |
|  | NIV / IV | 128(60.7%) | 86(39.3%) | | 0.985 |
|  | HFNC / NIV / IV | 185(59.7%) | 125(40.3%) | 0.999 | |
|  | Neutrophil count, X10^9^/L; median(IQR) | 7.7(4.6-12.4) | 9(3.8-14.6) | 0.362 | |
|  | Lymphocyte count, X10^9^/l; median(IQR) | 0.7(0.4-1.1) | 0.6(0.4-1.1) | 0.284 | |
|  | Haemoglobin concentration, g/L; median(IQR) | 90(81-101) | 90(83-98) | 0.622 | |
|  | Glucose concentration, mmol/L*; median(IQR) | 5.8(4.8-7) | 5.7(4.4-7.4) | 0.282 | |
|  | C-reactive Protein concentration, mg/L; median(IQR) | 220(124-305) | 208.5(122-305) | 0.770 | |
| **Throughout admission** | |  |  | |  |
|  | HFNC | 122(59.8%) | 82(40.2%) | | 0.991 |
|  | NIV | 64(59.8%) | 43(40.2%) | | 1 |
|  | NIV / IV | 159(60.7%) | 103(39.3%) | | 0.669 |
|  | HFNC /NIV/ IV | 206(59.5%) | 140(40.5%) | 1 | |

Supplementary table 2. Additional univariate analysis comparing survivors (n=353) and non-survivors (n=240) at day 90. Continuous variables were compared using Wilcoxon rank sum test. Categorical variables were compared using Chi Squared or Fisher’s Exact test. (LOS = length of stay, HFNC= High flow nasal cannulae, NIV= non-invasive ventilation, IV = invasive ventilation.) * = missing data for one patient.

**Supplementary table 3. Microbiological results from admission.**

| **Microbiology** | **Number of patients (n = 625), n (%)** |
| --- | --- |
| **Patients with positive tests (as % of patients, n = 625)** | |
| Positive blood culture | 132(21.1%) |
| Positive urine culture | 94(15.0%) |
| Positive sputum culture | 118(18.9%) |
| Any positive culture | 283(45.3%) |
| Positive galactomannan / beta-glucan | 35(5.6%) |
| Patients with positive PCR tests | 30(4.8%) |
| **Cultured organisms, n (as % of total organisms on blood culture, n = 173)** | |
| Fungal | 9(5.2%) |
| Candida sp. | 8(4.6%) |
| other | 1(0.6%) |
| Gram negative bacteria | 101(58.4%) |
| Bacteroides sp. | 7(4.0%) |
| Citrobacter sp. | 6(3.5%) |
| Enterobacter sp. | 6(3.5%) |
| Escherichia coli | 34(19.7%) |
| Klebsiella sp. | 27(15.6%) |
| Pseudomonas sp. | 10(5.8%) |
| other | 11(6.4%) |
| Gram positive bacteria | 63(36.4%) |
| Clostridium sp. | 6(3.5%) |
| Enterococcus sp. | 13(7.5%) |
| Staphylococcus sp. | 28(16.2%) |
| Streptococcus sp. | 6(3.5%) |
| other | 10(5.8%) |
| **PCR identified organisms, n (as % of positive PCRs, n = 50)** | |
| Ebstein Barr Virus | 18(36%) |
| Cytomegalovirus | 13(26%) |
| Herpes Simplex virus | 4(8%) |
| other viral | 11(22%) |
| Pneumocystis Carinii | 4(8%) |

Supplementary table 3. Microbiological results. Samples were obtained within 2 days before and 2 days following admission apart from PCR and beta-glucan or galactomannan which were collected between 1 week before and 2 weeks after ICU admission. (PCR = polymerase chain reaction)

**Supplementary table 4. Positive microbiology in survivors and non-survivors at day 90.**

| **Microbiology** | **D90 survivors, (n = 353), n (as % of patients with positive results)** | **D90 non-survivors (n=240), n (as % of patients with positive results)** | **p value** |
| --- | --- | --- | --- |
| Positive blood culture | 73(57.9%) | 53(42.1%) | 0.758 |
| Positive urine culture | 54(64.3%) | 30(35.7%) | 0.401 |
| Positive sputum culture | 59(54.6%) | 49(45.4%) | 0.299 |
| Positive stool culture | 11(64.7%) | 6(35.3%) | 0.849 |
| Any positive culture | 161(59.2%) | 111(40.1%) | 0.944 |
| Positive galactomannan / beta-glucan | 41(54.7%) | 34(45.3%) | 0.428 |
| Patients with positive PCR tests | 19(57.6%) | 14(42.4%) | 0.958 |

Supplementary table 4. The number of individuals with positive tests separated into survivors and non-survivors at day 90 is shown. The total number of tests in each category is less than in Supplementary table 3 as patients lost to follow up were not included in this analysis. There were no statistically significant differences. Variables were compared using Chi Squared or Fisher’s Exact test. (PCR = polymerase chain reaction)

**Supplementary table 5. Supplementary information on APACHE II and SOFA scores in survivors and non-survivors at day 90.**

| **Variable** | **Category** | **Day 90 survivors, (n = 353) n / % (% / IQR)** | **Day 90 non-survivors, (N = 240), n / % (% / IQR)** | **p value** |
| --- | --- | --- | --- | --- |
| % of total variables used in APACHE II calculation which are missing | | 0%(0-0) | 0%(0-0.04) | 0.033 |
|  |  |  |  |  |
| Cardiovascular SOFA | 0 | 69(19.5%) | 43(17.9%) | 0.122 |
|  | 1 | 148(41.9%) | 88(36.7%) |  |
|  | 3 | 50(14.2%) | 29(12.1%) |  |
|  | 4 | 86(24.4%) | 80(33.3%) |  |
|  |  |  |  |  |
| Liver SOFA | 0 | 221(62.6%) | 129(53.8%) | <0.001 |
|  | 1 | 80(22.7%) | 44(18.3%) |  |
|  | 2 | 42(11.9%) | 49(20.4%) |  |
|  | 3 | 9(2.5%) | 11(4.6%) |  |
|  | 4 | 1(0.3%) | 7(2.9%) |  |
|  |  |  |  |  |
| Renal SOFA | 0 | 247(70.0%) | 145(60.4%) | <0.001 |
|  | 1 | 59(16.7%) | 38(15.8%) |  |
|  | 2 | 30(8.5%) | 15(6.3%) |  |
|  | 3 | 12(3.4%) | 23(9.6%) |  |
|  | 4 | 5(1.4%) | 19(7.9%) |  |
|  |  |  |  |  |
| Coagulation SOFA | 0 | 273(77.3%) | 137(57.1%) | <0.001 |
|  | 1 | 43(12.2%) | 39(16.3%) |  |
|  | 2 | 25(7.1%) | 34(14.2%) |  |
|  | 3 | 9(2.5%) | 25(10.4%) |  |
|  | 4 | 3(0.8%) | 5(2.1%) |  |
|  |  |  |  |  |
| Neurological SOFA | 0 | 179(50.7%) | 127(52.9%) | 0.41 |
|  | 1 | 74(21.0%) | 43(17.9%) |  |
|  | 2 | 19(5.4%) | 8(3.3%) |  |
|  | 3 | 10(2.8%) | 12(5.0%) |  |
|  | 4 | 71(20.1%) | 50(20.8%) |  |
|  |  |  |  |  |
| Respiratory SOFA | 0 | 46(13.0%) | 35(14.6%) | 0.37 |
|  | 1 | 67(19.0%) | 32(13.3%) |  |
|  | 2 | 192(54.4%) | 136(56.7%) | |
|  | 3 | 33(9.3%) | 22(9.2%) |  |
|  | 4 | 15(4.2%) | 15(6.3%) |  |

Supplementary table 5. Additional information on APACHE II and SOFA scores in survivors and non-survivors at day 90. There was a statistically significant difference in the percent of missing APACHE variables in survivors and non-survivors ( p = 0.033, wilcoxon rank sum). There were no statistically significant differences in the score of the cardiovascular, respiratory and neurological components of the SOFA score between survivors and non-survivors. There was a statistically significant difference in the renal, liver and coagulation components of the SOFA score between survivors and non-survivors at day 90. Continuous variables were compared using Wilcoxon Rank Sum test. Categorical variables were compared using Chi Squared or Fisher’s Exact test. (SOFA = Sequential Organ Failure Assessment Score, APACHE II = Acute Physiology and Chronic Health Evaluation II.)

**Supplementary table 6. Variables considered in multivariate analysis.**

| **Variables considered in multivariate analysis** |
| --- |
| SEER summary stage |
| One year survival of underlying cancer |
| Year |
| Surgery on day of admission |
| Albumin |
| Radiotherapy |
| SOFA |
| Lactate |
| Systemic Anti-Cancer Treatment |
| Renal replacement |
| Septic shock |
| Neutropenia |
| Age |
| Gender |
| Lymphocytes |
| Haemoglobin |
| Glucose |
| CRP |
| Hospital LOS before admission |
| Bacteraemia |

Supplementary table 6. Variables considered in multivariate analysis. These variables were considered as they are features present at admission and attempt to limit co-linearity by providing mainly unique information. All the variables listed were input into forward selection to generate the model shown in table 3. Year was split into 3 equal groups to limit the number of variables in multivariate analysis (2010-2014, 2015-2017 and 2018-2020). (SOFA = Sequential Organ Failure Assessment Score, SEER = Surveillance, Epidemiology and End Results, CRP = C-reactive protein.)

**Supplementary table 7. Sensitivity analyses.**

| **Variable** | | **Day 90 survivors (n=353), n or median (%/ IQR)** | **Day 90 non-survivors (n = 240), n or median (%/ IQR)** |
| --- | --- | --- | --- |
| **respiratory SOFA score of 0** | | 46(13.0%) | 35(14.6%) |
|  | calculated PF ratio | 19(5.4%) | 15(6.25%) |
|  | unable to calculate PF ratio | 27(7.6%) | 20(8.3%) |
| **Patients with APACHE II score calculated without any missing values** | | 261(73.9%) | 164(68.3%) |
| APACHE II score in these patients only | | 19(16-23) | 21(17-26) |

Supplementary table 7. In SOFA score calculation, a respiratory score of 0 is assigned to individuals with a PF ratio of over 400mmHg necessitating arterial blood sampling. In our study, patients allocated a respiratory SOFA score of 0 included individuals where we were able to calculate a PF ratio and this was over 400mmHg and individuals without an arterial blood gas sample where we were unable to calculate a PF ratio but assumed normal physiological values. We undertook sensitivity analysis to assess whether individuals in this group had different 90 day survival depending on whether they had arterial blood sampling or not. There was no statistically significant difference in the % of survivors and non-survivors at day 90 when comparing individuals allocated a respiratory SOFA score of 0 based on a calculated PF ratio versus individuals where we were unable to calculate a PF ratio. Considering only patients without any missing variables in the calculation of APACHE II score led to a slight increase in the median score (by one) in both survivors and non-survivors at day 90. SOFA = Sequential Organ Failure Assessment Score, II = Acute Physiology and Chronic Health Evaluation II.)

**Supplementary table 8. Multivariate sensitivity analysis.**

| Covariate |  | Acceleration Factor | 95% CI | p value |
| --- | --- | --- | --- | --- |
| SEER Summary Stage | Distant | - | - | - |
|  | Localised | 0.14 | (0.07-0.27) | <0.001 |
|  | Regionalised | 0.22 | (0.13-0.38) | <0.001 |
| SOFA |  | 1.19 | (1.12-1.27) | <0.001 |
| Lactate |  | 1.27 | (1.16-1.37) | <0.001 |
| Surgery on day of admission | No | - | - | - |
|  | Yes | 0.13 | (0.07-0.28) | <0.001 |
| Albumin |  | 0.94 | (0.90-0.97) | <0.001 |
| Bacteraemia | No | - | - | - |
|  | Yes | 0.46 | (0.27-0.79) | 0.002 |
| Radiotherapy | No | - | - | - |
|  | Yes | 1.82 | (1.09-3.03) | 0.010 |
| Age |  | 1.02 | (1.00-1.04) | 0.006 |
| Medical Cancer Treatment | No | - | - | - |
|  | Yes | 1.54 | (0.96-2.44) | 0.040 |

Supplementary table 8. Multivariate analysis for an accelerated failure time model identifying the best predictors of 90-day survival excluding national one year survival of underlying cancer. P values were calculated using the Wald test. (SOFA = Sequential Organ Failure Assessment Score, SEER = Surveillance, Epidemiology and End Results.)

**Supplementary table 9. Sensitivity analysis: Binary logistic regression for the presence of death or survival at day 90.**

| Covariate |  | Odds Ratio | 95% CI | p value |
| --- | --- | --- | --- | --- |
| SEER Summary Stage | Distant | - | - | - |
|  | Localised | 0.13 | 0.07-0.25 | <0.001 |
|  | Regionalised | 0.18 | 0.11-0.30 | <0.001 |
| SOFA |  | 1.12 | 1.05-1.19 | <0.001 |
| Lactate |  | 1.11 | 1.02-1.22 | 0.015 |
| Surgery on day of admission | No | - | - | - |
|  | Yes | 0.20 | 0.09-0.41 | <0.001 |
| Albumin |  | 0.94 | 0.90-0.98 | 0.002 |
| National one year survival of underlying cancer |  | 0.99 | 0.98-0.99 | 0.002 |
| Bacteraemia | No | - | - | - |
|  | Yes | 0.64 | 0.38-1.06 | 0.086 |
| Radiotherapy | No | - | - | - |
|  | Yes | 1.69 | 1.03-2.80 | 0.038 |
| Age |  | 1.01 | 1.00-1.03 | 0.090 |
| Year of admission | 2010-2014 | - | - | - |
|  | 2015-2017 | 1.41 | 0.86-2.32 | 0.177 |
|  | 2018-2020 | 2.14 | 1.29-3.58 | 0.003 |
| Renal Replacement therapy on admission | No | - | - | - |
|  | Yes | 2.55 | 1.02-6.93 | 0.054 |
| Medical Cancer Treatment | No | - | - | - |
|  | Yes | 1.44 | 0.92-2.28 | 0.113 |

Supplementary table 9. Sensitivity analysis: Binary logistic regression for the presence of death or survival at day 90 following ICU admission. The factors above were identified by stepwise logistic regression. (SOFA = Sequential Organ Failure Assessment Score, SEER = Surveillance, Epidemiology and End Results, n = 589 patients).

**Supplementary References**

1. Lambden S, Laterre PF, Levy MM, Francois B. The SOFA score—development, utility and challenges of accurate assessment in clinical trials. *Crit Care*. 2019;23(1):374. doi:10.1186/s13054-019-2663-7

2. Vincent JL, Moreno R, Takala J, et al. The SOFA (Sepsis-related Organ Failure Assessment) score to describe organ dysfunction/failure. *Intensive Care Med*. 1996;22(7):707-710. doi:10.1007/BF01709751

3. Shah AD, MacCallum NS, Harris S, et al. Descriptors of Sepsis Using the Sepsis-3 Criteria: A Cohort Study in Critical Care Units Within the U.K. National Institute for Health Research Critical Care Health Informatics Collaborative. *Crit Care Med*. 2021;49(11):1883-1894. doi:10.1097/CCM.0000000000005169

4. National Disease Registration Service. *The National Cancer Registration and Analysis Service Routes to Diagnosis 2006-2016 Workbook* .; 2016. Accessed July 20, 2022. https://www.cancerdata.nhs.uk/routestodiagnosis/Routes_to_Diagnosis_2006_2016_workbook_published_version_a.xlsx
